# Trends and determinants of Comprehensive HIV and AIDS knowledge among urban young women in Kenya

**DOI:** 10.1186/1742-6405-8-11

**Published:** 2011-03-04

**Authors:** Rhoune Ochako, Dunstone Ulwodi, Purity Njagi, Steven Kimetu, Aggrey Onyango

**Affiliations:** 1 African Population and Health Research Center (APHRC), P.O. Box 10787, 00100 Nairobi, Kenya; 2 Ministry of Finance, P.O. Box 30007-00100 Nairobi, Kenya; 3 Care International in Kenya, Box 43864 Nairobi; 4 Liverpool VCT, P.O. Box 19835-00202, KNH, Nairobi Kenya; 5 Advanced Initiatives for Population & Development (AIPD), P.O. Box 6892, 00100 Nairobi, Kenya

## Abstract

**Background:**

Sub-Saharan Africa remains the region most heavily affected by HIV. In 2008, the region accounted for 67% of HIV infections worldwide, the region also accounted for 72% of the world's AIDS-related deaths in 2008. Young people aged 15-24 years accounted for an estimated 45% of the new HIV infections. In sub-Saharan Africa, Kenya is among countries affected by the HIV and AIDS pandemic which led to the declaration of AIDS as a national disaster in 1999. Given these scenario the study was undertaken to examine trends in HIV and AIDS comprehensive knowledge and identify the main correlates of comprehensive HIV and AIDS knowledge among Kenyan urban young women.

**Methods:**

Data used was drawn from the 1993, 1998, 2003 and 2008/09 Kenya Demographic & Health Surveys. Logistic regression was used for analysis.

**Results:**

While comprehensive HIV and AIDS knowledge is low among urban young women in Kenya, the results show a significant increase in comprehensive knowledge from 9% in 1993 to 54% in 2008/09. The strongest predictors for having comprehensive knowledge were found to be 1) education; 2) having tested for HIV; 3) knowing someone with HIV, and/or 4) having a small or moderate to great risk perception.

**Conclusion:**

The response to HIV and AIDS can only be successful if individuals adopt behaviours that will protect against infection. Currently, efforts are underway in Kenya to ensure that young people have comprehensive knowledge. As evident from the results, comprehensive HIV and AIDS knowledge has increased over the 15 year period among urban young women from 9% in 1993 to 54% in 2008/09. Despite this improvement, a lot more needs to be done to attain the target of 90% threshold set by UNGASS. While both young women and men should be targeted with education on HIV prevention, concerted efforts should be directed at young women as many continue to get infected due to low levels of comprehensive HIV knowledge.

## Background

Globally, sub-Saharan Africa (SSA) has been worst affected by HIV as it accounted for more than 68% the burden of the disease with more than 72% of all AIDS deaths recorded in 2008 [1,2]. New HIV infections were estimated at 1.7 million in 2007, accumulating to 22.5 million people living with the virus; of which, women accounted for 61% and young people aged 15-24 years accounted for an estimated 45% of the new HIV infections. In SSA region, Kenya is among countries worst affected by the AIDS pandemic [3], and this led to the declaration of AIDS as a national disaster in 1999. Since then, the National AIDS Control Council (NACC) was established to coordinate resources for prevention of HIV transmission and provision of care and support to the infected and affected [4]. Currently, about 1.4 million people in are infected with HIV, and women continue to be disproportionately infected having a prevalence of 8.7% compared to 5.6% among men [4]. Compared to young men, women aged 15-24, are 4 times more likely to be infected with HIV [4]. Monitoring of the the epidemic and assessing the impact of prevention, treatment and care programmes is done by the National AIDS Control Council (NACC) through the Kenya AIDS Indicator Survey (KAIS) [4].

In Kenya, the response to HIV and AIDS pandemic relies on preventive strategies where information on modes of transmission are provided to enable people identify and avoid risky behaviour that could expose them to infection [5]. Having accurate HIV and AIDS knowledge about transmission and prevention is important for avoiding HIV infection and ending the stigma and discrimination of infected and affected persons. Over 90% of the Kenyan population have heard about HIV and AIDS [6]. However, comprehensive HIV and AIDS knowledge levels among young people compares to that of other SSA countries where on average, about 30% of males and 19% of females aged 15-24 have accurate knowledge about HIV and avoiding its transmission. This is below the target set in 2001 by the United Nations General Assembly Special Session (UNGASS) to ensure 90% of young people aged 15-24 worldwide have comprehensive HIV and AIDS knowledge. Comprehensive knowledge means a person can correctly identify the two major methods of preventing the sexual transmission of HIV (using condoms and limiting sex to one faithful, uninfected partner), reject the two most common local misconceptions of HIV transmission in Kenya and knows that a healthy looking individual could have HIV [7].

Rapid urbanization in Kenya has presented development challenges leading to deteriorating living conditions and growing urban poverty [8]. Young people form a large proportion of those moving from rural to urban areas in search of livelihood opportunities, in the process most find urban slums as the first entry points into the cities. This present enormous challenges as most of these urban slums are underserved by health facilities, and challenged by other socio-economic amenities [9,10]. Because of limited livelihood opportunities and the frustrations of unemployment, many young adults in these settings turn to risky sexual behaviours, they also seek comfort in prostitution and drug abuse which expose them to HIV. Other behavioral factors that increase young urban women's risk for HIV infection include having older sexual partners, inconsistent condom use, forced sex, and transactional sex [11]. While specific programs meet the needs of women and children, there remains a poor understanding of the reproductive health needs of young women [12]. In Ethiopia a study focusing on urban slum dwelling young women found them to be vulnerable to reproductive health problems including HIV and physiological susceptibility to heterosexual transmission [13,14]. Another study in Lesotho found sexual and physical violence to be key determinants to the country's severe HIV epidemic with both men and women believing that women have no right to refuse sexual abuse by their partners [15]. Given the increased vulnerability to HIV that young women in SSA face due to the aforementioned cultural, behavioural, and physiological factors, it is not surprising that several studies in Kenya and Tanzania estimated young women to be three to six times more likely to have HIV compared with males of the same age [4,16,17].

As would be expected, research shows that young women lack accurate and complete information on how to avoid exposure to HIV [18]. This study focused on the more disadvantaged group, young women, by looking at the trends and determinants of HIV and AIDS comprehensive knowledge among those who reside in urban areas and are aged 15-24 years. Using data from the 1993, 1998 2003 and 2008/2009 Kenya Demographic and Health Surveys (KDHS) this study addressed the following objectives:

1) To examine trends in HIV and AIDS comprehensive knowledge among young women in urban Kenya;

2) To identify the main correlates of comprehensive HIV and AIDS knowledge among urban young women.

## Data and methods

### Source of data

This study used data from the publicly available 1993, 1998, 2003 and 2008/2009 Kenya Demographic and Health Survey (KDHS) which are nationally representative surveys of women aged 15-49 years. The surveys provide data on demographic and health indicators to promote analysis on health and nutrition of women and children in developing countries. The KDHS applies probability sampling to provide nationally representative samples of women in the reproductive age (15-49 years). The Demographic and Health Surveys provide nationally representative data with particular focus on ensuring representativeness based on age, urban or rural residence and sex among other characteristics. The surveys are conducted by Measure DHS and the Kenya National Bureau of Statistics. The response rates for the 1993, 1998, 2003 and 2008/2009 KDHS were between 94% and 98%. The data was weighted in order to adjust for differences in probability of selection and to adjust for non-response. This study used data from 1993, 1998, 2003 and 2008/2009, to provide the trend in comprehensive HIV and AIDS knowledge among young women. As of December 2010, the latest survey data available was KDHS 2008/2009; bivariate and multivariate analysis was therefore based on the latest available data, KDHS 2008/2009, to provide a clear indication on the most recent determinants of HIV and AIDS comprehensive knowledge among urban young women. The analysis is based on the urban sample [19] -defined by attributes like population size and density, administrative function, availability of social amenities and physical infrastructure such as hospitals, post office, schools and markets- of women aged 15-24. Young women are those within this age bracket as adopted and applied by the United Nations, World Bank and the Government of Kenya [20,21].

### Study variables

The dependent variable is a score, *comprehensive HIV and AIDS knowledge*, defined as correct knowledge of two ways to prevent HIV and rejection of three misconceptions about HIV. To measure comprehensive HIV and AIDS knowledge, each woman was asked whether or not she agreed or disagreed with the following five statements: 1) condoms can be used to prevent HIV transmission; 2) HIV can be prevented by limiting sex to one faithful uninfected partner; 3) a person can get HIV from mosquito bites; 4) a person can get HIV by sharing a meal with someone infected and 5) a healthy looking person can have HIV. Based on similar studies in Ethiopia and Kenya [5,22], the independent variables used in this paper include education (coded as none, primary and secondary/higher), household wealth (recoded as tertiles and labelled poor, middle and rich), ethnicity, parity, age, marital status, religion, and region of residence. Other factors included HIV and AIDS risk perception (measured by thoughts on her chances of getting AIDS: none, small, moderate/great), whether the respondent has gone for HIV and AIDS testing and counselling, and whether the respondent personally knew somebody who has/had died of AIDS.

### Methods of analysis

The data was weighted during analysis to adjust for differences in probability of selection and non-response. The statistical software STATA version 10 was used for analysis. To achieve the first objective, descriptive statistics and logistic regression of the dependent variable and time (year of survey) were used. Bivariate and multivariate logistic regression was used to identify the main correlates of comprehensive HIV and AIDS knowledge (Objective 2). Explanatory variables were included in the multivariate model. Logistic regression was used since the dependent variable, a score of comprehensive HIV and AIDS knowledge, was constructed to be a binary outcome. The binary outcome was defined as; yes, if the respondent answered all five questions about HIV and AIDS correctly, and no, if the respondent had any incorrect answers. This is in line with the accepted definition of comprehensive HIV and AIDS knowledge as used widely and also adopted by this study.

## Results

### Sample description

Table 1 shows the description of 1,103 young women aged 15-24 from urban Kenya interviewed in the 2008/2009 survey. Slightly more than half, 54% of the respondents had comprehensive HIV and AIDS knowledge while 44% had primary level education. As expected, the sampled women were distributed almost equally in the three household wealth categories. With regard to ethnic affiliation, about 20% of women were Kikuyu, 16% were Luhya and 20% were Luo which reflect the major ethnic groups of Kenya as a whole. Majority of the urban young women, 59%, had no children, while about 61% were aged 20-24 years. Forty eight percent considered themselves at a small risk of acquiring HIV, and about 71% knew somebody who died of or had AIDS. Sixty percent of the young urban women had tested for HIV.

**Table 1:** Distribution of urban young women, 15-24, Kenya 2008/2009

Characteristics	%	Number of cases
**Comprehensive HIV and AIDS knowledge**		
No	46.1	508
Yes	53.9	595
**Region**		
Central	5.6	62
Nairobi	33.6	371
Coast	20.8	229
Eastern	2.7	30
Nyanza	12.7	140
Rift Valley	8.5	94
Western	9.6	106
North Eastern	6.5	71
**Ethnicity**		
Kikuyu	19.8	218
Luhya	15.6	172
Luo	19.5	215
Other	45.2	498
**Religion**		
Catholic	18.4	203
Protestant	61.4	677
Other	20.2	223
**Education**		
None	6.0	66
Primary	44.3	489
Secondary or higher	49.7	548
**Household wealth**		
Poor	33.4	368
Medium	33.4	368
Rich	33.2	367
**Marital status**		
Never married	60.0	662
Ever married	40.0	441
**Age**		
15-19	39.3	433
20-24	60.7	670
**Parity**		
0	59.0	651
1	24.8	274
2+	16.2	178
**Tested for HIV and AIDS**		
No	39.8	439
Yes	60.2	664
**HIV risk perception**		
No risk	7.4	81
Small	48.3	533
Moderate/great	44.3	489
**Know somebody who has/died of AIDS**		
No	28.6	315
Yes	71.4	788
**Total (N)**	**100.0**	**1103**

### Bivariate and multivariate analysis

Table 2 presents regression bivariate and multivariate analysis results of comprehensive HIV and AIDS knowledge among young women in Kenya. Bivariate results show that women from North Eastern were 56% less likely (p < 0.05), while those from Nairobi were more than 2.5 times more likely (p < 0.01) to have comprehensive HIV and AIDS knowledge compared to their Central province counterparts. On the other hand, multivariate results show that women from Nairobi and Coast were more than 2.4 and 2.7 times respectively more likely to have comprehensive HIV and AIDS knowledge compared to their counterparts from Central province (p < 0.01). Considering education, women with primary and at least secondary education were more than 6.8 and 17.5 times respectively more likely to have comprehensive HIV and AIDS knowledge than their counterparts without education (p < 0.01) in the bivariate model. These effects slightly reduced in the multivariate model where women with primary and at least secondary education were more than 4.8 and 9.5 times more likely to have comprehensive HIV and AIDS knowledge than those with no education (p < 0.01).

**Table 2:** Odds ratio, of comprehensive HIV and AIDS knowledge among young women in Kenya 2008/2009

Characteristic	Bivariate			Multivariate		
	**OR**	**95% CI**	***p***	**OR**	**95% CI**	***p***
**Region**						
Central	1.00			1.00		
Nairobi	2.46	(1.43-4.25)	0.001	2.42	(1.30-4.52)	0.005
Coast	1.53	(0.87-2.69)	0.140	2.72	(1.37-5.42)	0.004
Eastern	1.94	(0.80-4.72)	0.141	2.17	(0.81-5.80)	0.122
Nyanza	1.54	(0.84-2.81)	0.160	2.19	(1.05-4.58)	0.037
Rift Valley	1.24	(0.65-2.37)	0.510	1.51	(0.74-3.10)	0.256
Western	0.88	(0.47-1.67)	0.705	1.70	(0.79-3.66)	0.177
North Eastern	0.44	(0.21-0.92)	0.028	1.32	(0.53-3.31)	0.556
**Ethnicity**						
Kikuyu	1.00			1.00		
Luhya	0.54	(0.36-0.82)	0.003	0.66	(0.40-1.11)	0.119
Luo	0.72	(0.49-1.06)	0.092	0.67	(0.41-1.10)	0.112
Other	0.56	(0.40-0.77)	0.0001	0.76	(0.49-1.16)	0.203
**Religion**						
Catholic	1.00			1.00		
Protestant	1.29	(0.94-1.77)	0.112	1.10	(0.78-1.55)	0.593
Other	0.48	(0.33-0.71)	0.0001	0.74	(0.44-1.24)	0.255
**Education**						
None	1.00			1.00		
Primary	6.78	(3.04-15.14)	0.0001	4.75	(2.04-11.07)	0.0001
Secondary or higher	17.52	(7.84-39.13)	0.0001	9.54	(4.03-22.60)	0.0001
**Household wealth**						
Poor	1.00			1.00		
Medium	1.64	(1.22-2.19)	0.001	1.11	(0.80-1.54)	0.546
Rich	2.60	(1.93-3.50)	0.0001	1.25	(0.84-1.88)	0.276
**Marital status**						
Never married	1.00			1.00		
Ever married	0.71	(0.55-0.90)	0.005	0.77	(0.52-1.14)	0.184
**Age**						
15-19	1.00			1.00		
20-24	1.57	(1.23-2.00)	0.0001	1.37	(1.01-1.86)	0.044
**Parity**						
0	1.00			1.00		
1	0.89	(0.67-1.18)	0.421	1.04	(0.69-1.57)	0.843
2+	0.63	(0.45-0.88)	0.006	1.08	(0.64-1.80)	0.780
**Tested for HIV and AIDS**						
No	1.00			1.00		
Yes	1.60	(1.25-2.04)	0.0001	1.46	(1.05-2.04)	0.024
**HIV risk perception**						
No risk	1.00			1.00		
Small	2.00	(1.23-3.24)	0.005	2.10	(1.22-3.59)	0.007
Moderate/great	2.22	(1.37-3.61)	0.001	1.86	(1.08-3.18)	0.024
**Know somebody who has/died of AIDS**						
No	1.00			1.00		
Yes	1.51	(1.16-1.96)	0.002	1.10	(0.81-1.49)	0.540
**Total (N)**	**1103**			**1103**		

Bivariate results indicate that ever married women were less likely to have comprehensive HIV and AIDS knowledge (p < 0.01) than their never married counterparts but disadvantage disappears in the multivariate model. Women aged 20-24 years were 1.6 times more likely (p < 0.01) to have comprehensive knowledge than their counterparts aged 15-19. This advantage is confirmed further in the multivariate model where they were 1.4 times more likely to have comprehensive knowledge (p < 0.05). Women with two or more children were 37% less likely (p < 0.01) to have comprehensive knowledge compared to their counterparts with no children. Although the multivariate results showed they had an advantage over their counterparts with no children, their differences do not attain any statistical significance. Belonging to medium and rich households made the women be 1.6 and 2.6 times respectively more likely to have comprehensive HIV and AIDS knowledge (p < 0.01) compared to those from poor households. Surprisingly, this advantage did not attain statistical significance in the multivariate model.

Women from other religious groups were 52% less likely (p < 0.01) to have comprehensive knowledge than their Catholic counterparts although this disadvantage disappears in the multivariate model. Looking at ethnic affiliation, Luhya (46%), Luo (28%) and women from other ethnic groups (44%) were less likely to have comprehensive HIV and AIDS knowledge compared to their Kikuyu counterparts. However, these differences did not attain statistical significance in the multivariate model. Women who believed they had small or moderate/great risk to contracting HIV were more than two times more likely to have comprehensive knowledge that those who believed they had no risk (p < 0.01). This advantage is confirmed in the multivariate model. Women who had tested for HIV were 1.6 times more likely to have comprehensive HIV and AIDS knowledge than their counterparts who had not tested (p < 0.01). This advantage is confirmed in the multivariate model where they were again 1.5 times (p < 0.05) more likely to have comprehensive knowledge than their counterparts who had not tested. Young women who knew somebody who had or had died of AIDS were 1.5 times more likely to have comprehensive HIV and AIDS knowledge than their counterparts who knew nobody, this advantage although apparent in the multivariate model, did not attain any statistical significance. Based on the Odds ratios presented in Table 2, the strongest predictors for having comprehensive knowledge are 1) education, 2) having tested for HIV, 4) knowing someone with HIV, and/or 5) having a small or moderate/great risk perception. These findings will form the focus of our discussion.

### Trends in comprehensive HIV and AIDS knowledge

**Figure 1:**
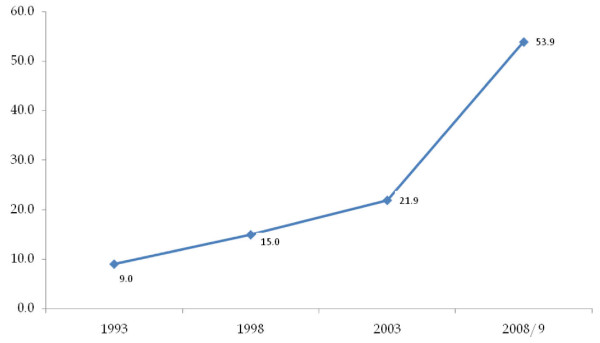
**Urban young women 15-24 with Comprehensive HIV and AIDS knowledge in Kenya**.

Survey data from 1993, 1998, 2003 and 2008/2009 reveal an increasing trend in comprehensive HIV and AIDS knowledge among young women resident in urban Kenya. The results indicate that the percentage of young urban women with comprehensive HIV and AIDS knowledge increased from 9% to 15% between 1993 and 1998 then to 22% in 2003 and further increased to 54% in 2008/09. These results are further illustrated in Figure 1. The interaction between time (year of survey) and comprehensive HIV and AIDS knowledge showed a 77% (p < 0.01) increase in comprehensive HIV and AIDS knowledge between survey 1 (1993) and 2 (1998). Young urban women in survey 3 (2003) were more than 2.8 times more likely to have comprehensive HIV and AIDS knowledge compared to those in survey 2. Similarly young urban women interviewed in survey 4 (2008/2009) were more than 11.8 times more likely to have comprehensive HIV and AIDS knowledge compared to their counterparts in survey 3. These results are further shown in Table 3.

**Table 3:** Interaction between time and comprehensive HIV and AIDS knowledge among young women, 1993-2008/2009

Characteristic			
	**OR**	**95% CI**	***p***
**Time**			
1993	1.00		
1998	1.77	(1.24-2.53)	0.002
2003	2.83	(2.06-3.89)	0.000
2008/09	11.80	(8.65-16.11)	0.000

## Discussion

This paper examines trends and determinants of comprehensive HIV and AIDS knowledge among urban young women in Kenya. Descriptive results on trends indicate that 54% of the urban young women had comprehensive HIV and AIDS knowledge, indicating an increase over the 15 year period from 9% in 1993. Although progress has been attained in terms of increase in comprehensive HIV and AIDS knowledge, this is still way below the 90% target set by UNGASS. General awareness of HIV and AIDS is high in Kenya, but awareness alone is not adequate for prevention. Rather, accurate and high levels of comprehensive knowledge on HIV and AIDS transmission is necessary [22,23]. The increasing trend in HIV and AIDS comprehensive knowledge among urban young women could be attributed to the increase in interventions targeting young people, especially young women. Such efforts are spearheaded by the government, institutions of learning and civil society organizations. According to study findings education plays a significant role in determining one's social status, and in many cases, it translates to better occupation, income and access to information [24]. This study found education to be a significant predictor of having comprehensive HIV and AIDS knowledge, a finding consistent with those of the 2007 Kenya AIDS Indicator Survey (KAIS) that also observed an increase in comprehensive HIV and AIDS knowledge among people with more years of education [4]. In a study among Malawian women, O'Fallon *et al. *(2004) found women with no education slightly less knowledgeable about HIV and AIDS compared to those with secondary or higher education [25]. Formal education may influence HIV and AIDS knowledge by not only providing young people with the information needed to protect themselves from infection, but by also motivating young people to take better care of their health for successful and prosperous future [26].

A sub set of ever married women had less comprehensive HIV and AIDS knowledge compared to their never married counterparts. Even though other studies have suggested that married women are unlikely to negotiate for safer sex and may be unaware of extra-marital affairs of their husbands, ever married women are likely have assumptions that marriage is protective of risk of infection and may assume they will benefit from their husbands knowledge of HIV and AIDS [22]. Notably, wealth, a proxy for social status, did not influence comprehensive HIV and AIDS knowledge; this may be due in part to the association of wealth with education given the dilution effect of wealth in the multivariate model. Although this study found young protestant women to have an advantage in terms of comprehensive HIV and AIDS knowledge over their Catholic counterparts, their differences did not attain any statistical significance both in the bivariate and multivariate models. On the contrary, a study in Mozambique found protestant women to have more comprehensive HIV and AIDS knowledge than their Catholics counterparts [27].

Young women who personally knew someone with or who had died of AIDS had more comprehensive knowledge than those not acquainted with affected individuals. Studies conducted in Malawi, Uganda, and Rwanda confirmed similar results revealing men and women acquainted with individuals with AIDS tended to have greater knowledge of HIV and AIDS and changed behavior due to their greater risk perception [25,28,29]. Young women with small or moderate/great risk perception were more likely to have comprehensive knowledge of HIV and AIDS than those who believed they were not at risk of contracting HIV. Although the number of people who know that HIV and AIDS exists is widespread, individual risk perception varies, and whether or not an individuals' risk perception is accurate, it may influence the adoption of risk reduction strategies [30]. Comprehensive HIV and AIDS knowledge was lower among those who had never tested for HIV.

## Limitations of the study

One limitation of the DHS is that, its sampling procedures to do not take into consideration the informal settlements although many slum residents are affected by HIV and AIDS.

## Conclusion

The response to HIV and AIDS can only be successful if individuals adopt behaviours that will protect against infection. Most HIV reduction strategies assume that when people are aware of the fatality of HIV and AIDS, they will adopt preventive measures to avoid infection and subsequent death. Currently, efforts are underway in Kenya to ensure that young people have comprehensive knowledge. As evident from the results, comprehensive knowledge has increased over the 15 year period among urban young women from 9% in 1993 to 54% in 2008/2009. Despite this improvement, a lot more needs to be to attain the target of 90% by UNGASS. The question is how much effort and time it will take to attain the set threshold. The 2007 KAIS found the prevalence of HIV between young women and men (15-24 years) to be 21.6% and 7.1% respectively. This further highlights the disadvantage young women face [4]. While both young women and men should be targeted with education on HIV and AIDS prevention, a lot more should be done to ensure more young women benefit as many of them continue to get infected due to lack of comprehensive knowledge on how to avoid HIV infection.

## Competing interests

The authors declare that they have no competing interests.

## Authors' contributions

RO: Participated in the inception of the idea of this manuscript, with lead roles in conducting literature review, data analysis, writing the results and discussion sections. DU and SK: prepared the background section, PN and AO: Prepared the discussion section. All authors read and approved the final manuscript.

## References

[B1] UNAIDS Key Facts by Region - 2007 AIDS Epidemic Update 2007 Geneva: UNAIDS

[B2] UNAIDS Report on the Global HIV/AIDS Epidemic. Joint United Nations Programme on HIV/AIDS 2007 Geneva: UNAIDS

[B3] K'Oyugi Boniface O Jane Muita The Impact of a Growing HIV/AIDS Epidemic on the Kenyan Children AIDS, PUBLIC POLICY AND CHILD WELL-BEING 2002 Florence: UNICEF Cornia Giovanni A ed

[B4] National AIDS/STI Control Programme 2007 Kenya AIDS indicator survey: final report 2009 Nairobi, Kenya: National AIDS/STI Control Programme

[B5] Zewdu Woubalem Half Baked HIV/AIDS Knowledge: Blessing or Curse? Journal of Health & Population in Developing Countries 2005

[B6] Munyisia E Marum LH Cheluget B Cherop M E W General and specific knowledge about HIV/AIDS among out of school youth in Kenya International Conference on AIDS 2004 Bangkok, Thailand

[B7] UNICEF Prevention of infection among adolescents and young people. Childinfo, Monitoring the situation of children and women 2009 UNICEF

[B8] Walter Odhiambo Damiano Kulundu Manda Urban poverty and labour force participation in Kenya 2003 Nairobi, Kenya: Kenya Institute for Public Policy Research and Analysis (KIPPRA); November

[B9] Environmental Health Project-USAID Improving the Health of the Urban Poor Learning from USAID Experience Strategic Report 12 2004

[B10] United Nations Population Fund Marshall A The State of World Population 1996: Changing Places: Population, Development and the Urban Future 1996

[B11] Pettifora Audrey E van der Stratena Ariane Dunbara Megan S Shiboskia SC Padiana Nancy S Early age of first sex: a risk factor for HIV infection among women in Zimbabwe 2004 Lippincott Williams &Wilkins 10.1097/01.aids.0000131338.61042.b8 15199320

[B12] Jejeebhoy Shireen J Adolescent Sexual and Reproductive behavior: A Review of the Evidence from India Social Science & Medicine 1998 46 1275 1290 10.1016/s0277-9536(97)10056-9 9665560

[B13] UNAIDS AIDS epidemic update 2009 Geneva, Switzerland: UNAIDS; November

[B14] Macro International HIV prevalence estimates from the demographic and health surveys 2008 Calverton, USA: Macro International

[B15] Andersson N Risk factors for domestic physical violence: national cross-sectional household surveys in eight southern African countries BMC Women's Health 2007 7 10.1186/1472-6874-7-11 PMC2042491 17631689

[B16] Tanzania Commission for AIDS Tanzania HIV/AIDS and malaria indicator survey 2007-2008 2008 Dar es Salaam: Tanzania Commission for AIDS

[B17] UNAIDS Listen, Learn, Live! World AIDS Campaign with Children and Young People: Facts and Figures 1999 Geneva: UNAIDS

[B18] UNAIDS Report on the global HIV/AIDS epidemic 2008 2008 Geneva: UNAIDS

[B19] Kenya National Bureau of Statistics (KNBS), ICF Macro Kenya Demographic and Health Survey 2008-09 2010 Calverton, Maryland: KNBS and ICF Macro

[B20] National Council for Population and Development Sessional Paper No. 1 of 2000 on National Population Policy for Sustainable Development 2000 Nairobi, Kenya: Ministry of Finance and Planning

[B21] United Nations Youth and the United Nations United Nations

[B22] Priscilla Akwara A Janet Madise Nyovani Hinde Andrew Perception of Risk of HIV/AIDS and sexual Behaviour in Kenya 2003 United Kingdom: Cambridge University Press

[B23] UNAIDS Programme Monitoring and Evaluation Indicators 2000 Geneva: UNAIDS

[B24] Rahman Mohammad Shafiqur, Rahman Mohammad Lutfor Media and education play a tremendous role in mounting AIDS awareness among married couples in Bangladesh AIDS Research and Therapy 2007 4 10.1186/1742-6405-4-10 PMC1877805 17498310

[B25] O'Fallon Barden Janine L deGraft-Johnson Joseph Bisika Thomas Sulzbach Sara Benson Aimee Tsui Amy O Factors Associated with HIV/AIDS Knowledge and Risk Perception in Rural Malawi AIDS and Behavior 2004 8 10.1023/B:AIBE.0000030244.92791.63 15187475

[B26] Eric Tenkorang Y Fernando Rajulton Eleanor Tyndale-Maticka Perceived Risks of HIV/AIDS and First Sexual Intercourse among Youth in Cape Town, South Africa AID behav 2009 13 234 245 10.1007/s10461-008-9470-5 18846419

[B27] Agadjanian V Gender, Religious involvement and HIV/AIDS prevention in Mozambique Social Science and Medicine 2005 61 1529 1539 10.1016/j.socscimed.2005.03.012 15869833

[B28] Ministry of Finance Uganda, Macro International Uganda Demographic & Health Survey 1997 Entebbe, Uganda and Calverton, MD

[B29] Bulterys M Chao A Habimana P Dushimimana A Nawrocki P Saah A Incident HIV-1 infection in a cohort of young women in Butare, Rwanda AIDS 1994 1585 1591 10.1097/00002030-199411000-00010 7848595

[B30] Hans-Peter Kohler Behrman Jere R Watkins Susan C Social Networks and HIV/AIDS Risk Perceptions Demography 2007 44 1 33 10.1353/dem.2007.0006 17461334

